# Analysis of the Protein Domain and Domain Architecture Content in Fungi and Its Application in the Search of New Antifungal Targets

**DOI:** 10.1371/journal.pcbi.1003733

**Published:** 2014-07-17

**Authors:** Alejandro Barrera, Ana Alastruey-Izquierdo, María J. Martín, Isabel Cuesta, Juan Antonio Vizcaíno

**Affiliations:** 1 European Molecular Biology Laboratory, European Bioinformatics Institute (EMBL-EBI), Wellcome Trust Genome Campus, Hinxton, Cambridge, United Kingdom; 2 National Centre for Microbiology, Instituto de Salud Carlos III, Majadahonda, Madrid, Spain; The Centre for Research and Technology, Hellas, Greece

## Abstract

Over the past several years fungal infections have shown an increasing incidence in the susceptible population, and caused high mortality rates. In parallel, multi-resistant fungi are emerging in human infections. Therefore, the identification of new potential antifungal targets is a priority. The first task of this study was to analyse the protein domain and domain architecture content of the 137 fungal proteomes (corresponding to 111 species) available in UniProtKB (UniProt KnowledgeBase) by January 2013. The resulting list of core and exclusive domain and domain architectures is provided in this paper. It delineates the different levels of fungal taxonomic classification: phylum, subphylum, order, genus and species. The analysis highlighted *Aspergillus* as the most diverse genus in terms of exclusive domain content. In addition, we also investigated which domains could be considered promiscuous in the different organisms. As an application of this analysis, we explored three different ways to detect potential targets for antifungal drugs. First, we compared the domain and domain architecture content of the human and fungal proteomes, and identified those domains and domain architectures only present in fungi. Secondly, we looked for information regarding fungal pathways in public repositories, where proteins containing promiscuous domains could be involved. Three pathways were identified as a result: lovastatin biosynthesis, xylan degradation and biosynthesis of siroheme. Finally, we classified a subset of the studied fungi in five groups depending on their occurrence in clinical samples. We then looked for exclusive domains in the groups that were more relevant clinically and determined which of them had the potential to bind small molecules. Overall, this study provides a comprehensive analysis of the available fungal proteomes and shows three approaches that can be used as a first step in the detection of new antifungal targets.

## Author Summary

Some fungi have become pathogenic to plants and in a lesser extent to animals. Under certain conditions their presence in the human body can prove a threat for human health, especially for immunocompromised patients. Yet, some fungi can also infect healthy individuals. The low sensitivity of the antifungal drugs available together with the clinically observed resistance of some fungi raises the demand for new alternative treatments. Proteins are biological molecules which perform essential functions within the living organisms. Many of those functions are attributed to the varying folded structure of each protein. These configurations are composed of functional units -also called domains- each one independently responsible for a fraction of the overall biological function. Understanding how the different block combinations are distributed across members of the same or similar families of organisms is important. For instance, exclusive domain combinations can hold particular acquired functions. Blocks displaying a high mobility can play major roles for the organism's survival. The biological goal of this study was to analyse the functional implications of protein domains and domain combinations in the available fungal proteomes. This information can be used to highlight proteins and pathways that could be potentially used as drug targets.

## Introduction

There has been a significant rise in the incidence of fungal infection over the last few years. This has been partially due to an increase in the susceptible population as the result of blood cancer, intensive care, solid organ transplantation, or chronic granulomatous disease, in addition to a growing number of patients receiving high doses of corticosteroids or other immunosuppressive treatments [1], [2]. The most common cause of invasive fungal infection (IFI) is *Candida* spp. followed by *Aspergillus* spp. [3], [4]. The mortality of candidemia ranges from 44% to 75% [1], [5] and that of aspergillosis is around 60% [4]. IFI constitutes even a more important problem for immunocompromised patients, with mortality reaching up to 90% [6]. It has also been observed that new fungal species are part of the etiology of these infections, the common characteristic being an enhanced resistance to known antifungal agents, which further complicates the management of these infections. The success of available antifungal therapies is limited by the drug toxicity for the host (resulting potentially in severe side effects) and the late detection and treatment of the infection [7]. The cost of patient hospitalization, due to the usual long period needed for the currently available drugs to be efficient, is another factor to consider. Therefore, the discovery of new drug targets and antifungal drugs with a broader spectrum of activity is one active field of research.

The rationale in the search for new antifungal targets in the pre-genomics era was based on the molecular study of genes associated with fungal viability or virulence. The advent of massive parallel sequencing technologies and their progressively reduced cost has enabled the sequencing of large numbers of genomes in a short period of time. As a result, it is now possible to look for broad-spectrum antifungal targets by detecting homologous proteins present in most of the available fungal proteomes [8]. Approaches based on sequence similarity (e.g. BLAST [Basic Local Alignment Search Tool]) have some difficulties when trying to detect homologous proteins present in distantly related species, when sequence similarity is less than 30% [9]. However, protein domain composition is likely to be conserved throughout evolution due to functional constraints [10], so it can be used for this purpose.

In the late 90s, the concept of protein domain was coined by Branden and co-workers to define an independent, compact and stable protein structural unit that folds independently of other such units [11]. Most proteins in eukaryotes are composed of different domains, which are independent units with potentially different biological functions. The term domain has also been used to name blocks of protein sequences highly conserved throughout the course of the evolution. The order in which these domains are arranged within the protein sequence constitutes its domain architecture. Proteins having the same domain architecture are likely to have similar structures and therefore preserve the same cellular function [12]. The emergence of proteins with new domain and/or domain combinations is thought to be a major mechanism of evolution since it can give rise to new functions for the organisms [13], [14]. In this context, the term ‘merology’ has been proposed to describe the fact that the different units of multidomain proteins have different evolutionary history. Correspondingly, ‘merologous’ proteins refer to non-homologous proteins that display the same domain organization [15].

Whereas the domain repertoires of different organisms can be relatively similar, there can be a high variation in the number of the domain combinations. Therefore, the number of different domain architectures is more variable and is related to the organism complexity and lifestyle [12], [16]. In fact, the emergence of animals and vertebrates has been associated with the appearance of novel domain combinations [13], [17]. Domain promiscuity is a measurement of the capability of a protein domain to combine with other domains. For instance, it has been shown that the high mobility of protein structural units embraces the capacity to interact with other proteins and might hold important roles in Protein-Protein Interaction (PPI) networks [18]. Intuitively, a protein domain to be considered promiscuous must appear in several domain combinations within a proteome. However, different studies have observed that the number of domain combinations correlates positively with the degree in which such domain is spread over a genome [11]. Nevertheless, most approaches used to analyse domain promiscuity normalize the number of distinct combinations by the number of times a domain is found in the genome.

Protein domain information can be used for many different purposes. For instance, the domain architecture can determine the overall protein function and be used to transfer genomic annotations in newly sequenced genomes [19], using automatic annotation approaches. Domain configurations can also be exploited to determine the phylogeny of complete genomes, achieving comparable results than those using more sophisticated methods [20]. Furthermore, protein domains can be studied in the context of their ability to bind small molecules, since they can represent targets for biologically important ligands including potential drugs. One example of this approach is a recent study [21] that predicted *in silico* the potential of ligands and small molecules included in the ChEMBL database [22] to bind protein domains included in Pfam [7]. The underlying assumption was that ligands and small molecules could bind structurally conserved protein regions, supported by the reported observation that 88.4% of annotated binding sites from UniProtKB/Swiss-Prot rested entirely within the boundaries of a given Pfam domain [21].

In this manuscript, we first perform an analysis of the protein domains and domain architectures derived from all the available complete fungal proteomes. We identify the core and exclusive domains and domain architectures at different levels of the fungal taxonomical classification (phylum, subphylum, order, genus, species) and characterize which domains are found to be promiscuous. Then, we use the obtained protein domain information to explore *in silico* approaches to detect potential candidate drug targets, using information coming from different public resources and previous studies. In addition, we also classify some fungal species according to their occurrence in clinical samples and provide the related exclusive domain/domain architecture related information.

## Results/Discussion

### 1. Analysis of protein domain content

Proteins from all the available complete fungal proteomes (137 organisms) were retrieved from UniProtKB (UniProt Knowledgebase, release 2013_01). The 137 fungal proteome sets represented 111 species of the following four fungal phyla: Ascomycota, Basidiomycota, Chytridiomycota and Zygomycota. In addition, the phylum Microsporidia was also considered. Microsporidia include a controversial group of eukaryotic organisms of fungal origin, without mitochondria and peroxisomes. Microsporidia have been considered in this study since they are considered the earliest-diverging clade of sequenced fungi [23] and some of them are causative agents of disease in immunocompromised humans [24]. Specifically, the vast majority of organisms analysed belonged to the Ascomycota phylum (112 proteomes, containing 88 different species) followed by Basidiomycota (18 proteomes, containing 16 species), Microsporidia (5 proteomes coming from the same number of species), and Chytridiomycota and Zygomycota, each of them with only one representative species. Proteomes from different strains of the same species were included in the study e.g. *Saccharomyces cerevisiae* (13 strains) or *Ajellomyces capsulata* (4 strains). For a complete list of species and strains, see Table S1. Overall, 1,191,070 proteins were analysed, with an average of 8,694 proteins per proteome.

The related domain information for each protein was obtained from Pfam (release 27.0). On average, 67.0% of the proteins had at least one Pfam domain assigned. However, the Pfam coverage was not evenly distributed among the studied species. On the low coverage side, there were plant pathogens like *Melampsora laricis-populina* (strain 98AG31/pathotype 3-4-7), the poplar leaf rust fungus, and *Puccinia graminis* sp. *tritici* (CRL 75-36-700-3/race SCCL), the causal agent of wheat and barley stem rust. These fungi only had a 36.5% and 39.5% of Pfam coverage, respectively. The likely explanation for this lower coverage is that some of the domains are not found in the traditional model organisms and therefore not included in Pfam. On the contrary, the fission yeast *Schizosaccharomyces pombe* (strain 972/ATCC 24843) and its close relative species *S. japonicus* (strain yFS275/FY16936) showed a coverage over 87.5%. The complete coverage information per organism can also be found in Table S1.

On average, each fungal protein in the studied set had 1.55 domains, a proportion that did not vary significantly between species. Overall, 5,279 different Pfam domains were found among the studied proteomes (35.6% of the total Pfam domains). Among them, 131 domains (2.5%) were found in every fungus analysed (they are ‘core’ domains), whereas 612 domains (11.6%) were found only in one organism. Interestingly, when Microsporidia organisms were not considered in the analysis, the number of fungal ‘core’ domains rose to 268. Overall, 70.8% of all domain types (3,740) appeared in both single and multidomain proteins. In addition, 964 domains (18.3%) were not seen in combination with any other domain, whereas 575 domains (10.9%) appeared exclusively in multidomain proteins (see Table S2 for the complete lists of domains in each category). The domain repertoire found in the different fungal species was similar, with the exception of some Microsporidia species, which contained proportionally fewer domain types (Table S1). No linear correlation was found between the number of distinct domains and the size of the proteome. From an evolutionary perspective this should not be surprising since larger proteomes can be explained by an increasing number of duplication events, creating new copies of the same domain [25].

### 2. Diversity and relationship of protein domains and protein domain architectures

Protein domain architectures were defined throughout this study as the ordered tuples of Pfam domains, listed from the protein N-terminus to C-terminus. Architectures with either different domain counts or order were computed as independent domain combinations, even if they had the same domain types. For instance, the representation of three potential different example domain architectures with just two domains could be: 1) D1∼D1∼D2; 2) D1∼D2; and 3) D1∼D2∼D1. As indicated, all three would be considered different domain architectures because either the number of domains, the order of the domains and/or the number of domains were different.

In total, 21,853 unique domain architectures were identified in our set of 137 fungal proteomes, with 10,206 of them (46.7%) appearing exclusively in a single proteome and only 56 ‘core’ architectures (0.2%) that were present in all the fungi. However, when Microsporidia species were not taken into account, the number of ‘core’ architectures almost doubled: 107 domain architectures were found. Gene Ontology (GO) term annotations were added to give an insight of their molecular function and/or biological activity (see ‘Methods’). Approximately one third of all Pfam domains (32%) had one or more GO terms associated.

Most of these 56 ‘core’ domain architectures (Table S3) were composed of only one domain: there were 50 single domain and 6 multidomain ‘core’ architectures. As expected, the corresponding proteins are related with essential and well-conserved regulatory mechanisms such as control of vesicle formation (Ras family), DNA binding, modelling patterns of anatomical development (Homeobox domain), RNA metabolism, nuclear transcription and pre mRNA splicing (DEAD/DEAH box helicases). DEAD/DEAH box proteins are highly conserved among a wide variety of species. While DEAD proteins participate in the translation initiation of the spliceosome, DEAH proteins are required in further stages of the splicing process like the transesterification, releasing the mRNA and degradation of some spliceosome complexes [26].

The effect of removing Microsporidia from the analysis of ‘core’ domains and architectures highlights how different Microsporidia organisms can be, when compared with the rest of the organisms analysed. To measure how significant this increase is, we compared it to the effect induced by removing the species from other phylum (Zygomycota). Note that the only strain from Zygomycota (*Rhizopus delemar*) had more proteins (16,968) than the total amount of proteins from all the Microsporidia strains together (11,973). When Zygomycota were removed, the number of ‘core’ domains rose just from 131 to 132, whereas the number of ‘core’ architectures did not change.

**Figure 1:**
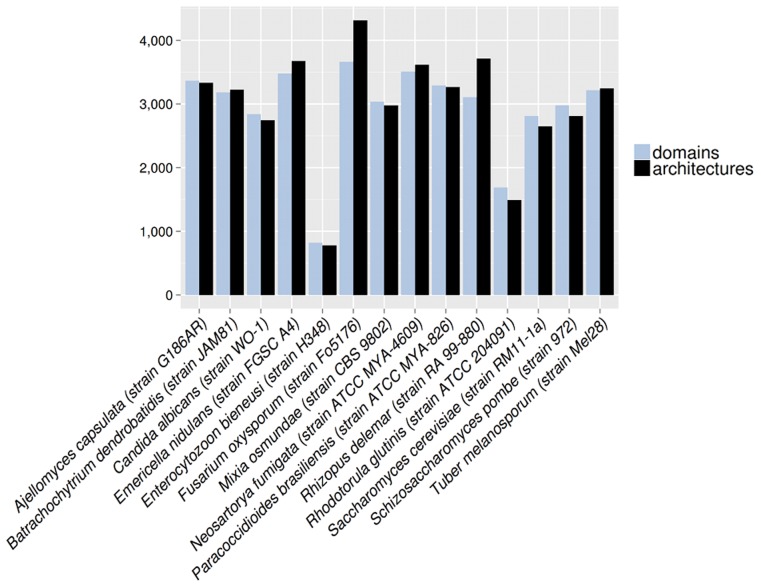
Distribution of number of Pfam domains and domain architectures found for selected species.

The same pattern of modest variance in the number of domains shown in the previous section was also observed at the level of the domain architectures (Table S1). On the contrary, the ratio between unique domains and unique domain architectures in a given species is more diverse across the fungal kingdom. We analysed the counts of distinct domains and domain architectures per proteome (Figure 1, for a more detailed representation see Figure S1). It was observed that filamentous fungi (such as *R. delemar, Fusarium oxysporum, Emericella nidulans or Neosartorya fumigata*) tend to have a larger ratio of architectures per domain, whereas yeasts (such as *Rhodotorula glutinis, S. cerevisiae or S. pombe*) in general hold less combinatorial power to create new architectures. Interestingly, the thermo-dimorphic (found in both filamentous and unicellular form) fungal pathogens *A. capsulata (Histoplasma capsulatum), Paracoccidioides brasiliensis* Pb01 (recently reclassified as *P. lutzii* [27], [28]) and *Candida albicans* (unicellular yeast also able to produce true hypha) were found in the middle section of the figure with an architecture/domain ratio value close to 1.

### 3. Exclusive domains and domain architectures per species and genus

At the species level, 637 domains and 10,582 domain architectures were identified as exclusive for one of the 111 fungal species analysed. In this case, proteomes from multiple strains belonging to the same species were grouped. Throughout the rest of the manuscript, organisms belonging to the phylum Microsporidia were considered as any other fungus. In any case, the different analyses were also performed without Microsporidia. However, the results are not shown for simplification purposes since we believe they do not add much novel information, unlike the information about ‘core’ domains and architectures, included in the previous sections.

**Figure 2:**
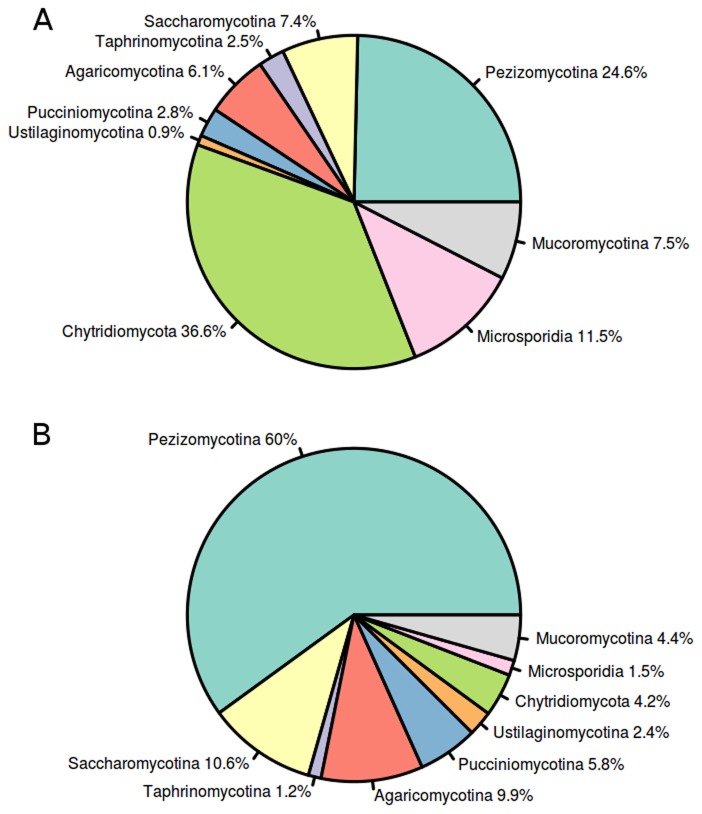
Distribution of protein domains (A) and domain architectures (B) exclusively found in the different fungal subphyla.

The average number of exclusive architectures per species was 96, ranging from 10 to 467 (see below). All the exclusive architectures for each species are provided as Table S4. In terms of subphyla, the 58 species from the Pezizomycotina subphylum contained an exceptional amount of exclusive domains (24.6%) and domain architectures (60.0%), as can be observed in Figure 2. Saccharomycotina, with 28 species, was also a very prominent group with a similar fraction of exclusive domains (7.4%) and domain architectures (10.6%). *Batrachochytrium dendrobatidis*, the only representative species of the Chytridiomycota subphylum had most of the exclusive domains (36.6%), but with a modest combinational power (only 4.2% of exclusive architectures).

The organisms with the least number of exclusive architectures were those from the primitive Microsporidia species *Encephalitozoon intestinalis* and *E. cuniculi*, with only 10 species-specific domain architectures. Then, there were two Saccharomycotina species: *Candida dubliniensis* (12 architectures) and *C. glabrata* (15 architectures). *Cryptococcus gattii serotype B* was the species with the lowest number of exclusive architectures in the phylum Basidiomycota (32 architectures).

As a simple observation, species without other closely related species in the dataset tended to have larger numbers of exclusive domain architectures. The highest numbers were found in *R. delemar* (467 architectures) and *Batrachochytrium dendrobatidis* (443), which were the only representatives of the Zygomycota and Chytridiomycota phyla, respectively. Remarkably, two Ascomycetes: the pathogen *F. oxysporum* (424) and *A. capsulata* (301), the causative agent of histoplasmosis [29], showed also a high number of exclusive architectures. Another prolific species in terms of exclusive architectures was *Mixia osmundae* (262), a Basidiomycete genetically highly divergent compared with other members of the subphylum Pucciniomycotina [30].

The repertoire of exclusive protein domains looked slightly different. For a complete list of exclusive domains per species see Table S5. On average, only 6 domains were found to be exclusive for one single species. Most species (94 out of 111) showed a smaller number and sixteen of them did not even account for a single exclusive domain. Among the species with the highest amount of exclusive domains were the already mentioned unique representatives for the phylum Chytridiomycota (*Batrachochytrium dendrobatidis*, 233 domains) and Zygomycota (*R. delemar*, 48). In addition, the Microsporidia member with the highest number of exclusive domains was *E. bienueusi*, with 68. Finally, the Ascomycete model organism *Sordaria macrospora* had 23, and the tree pathogen Basidiomycete *Moniliophthora perniciosa* had 11 exclusive domains.

**Figure 3:**
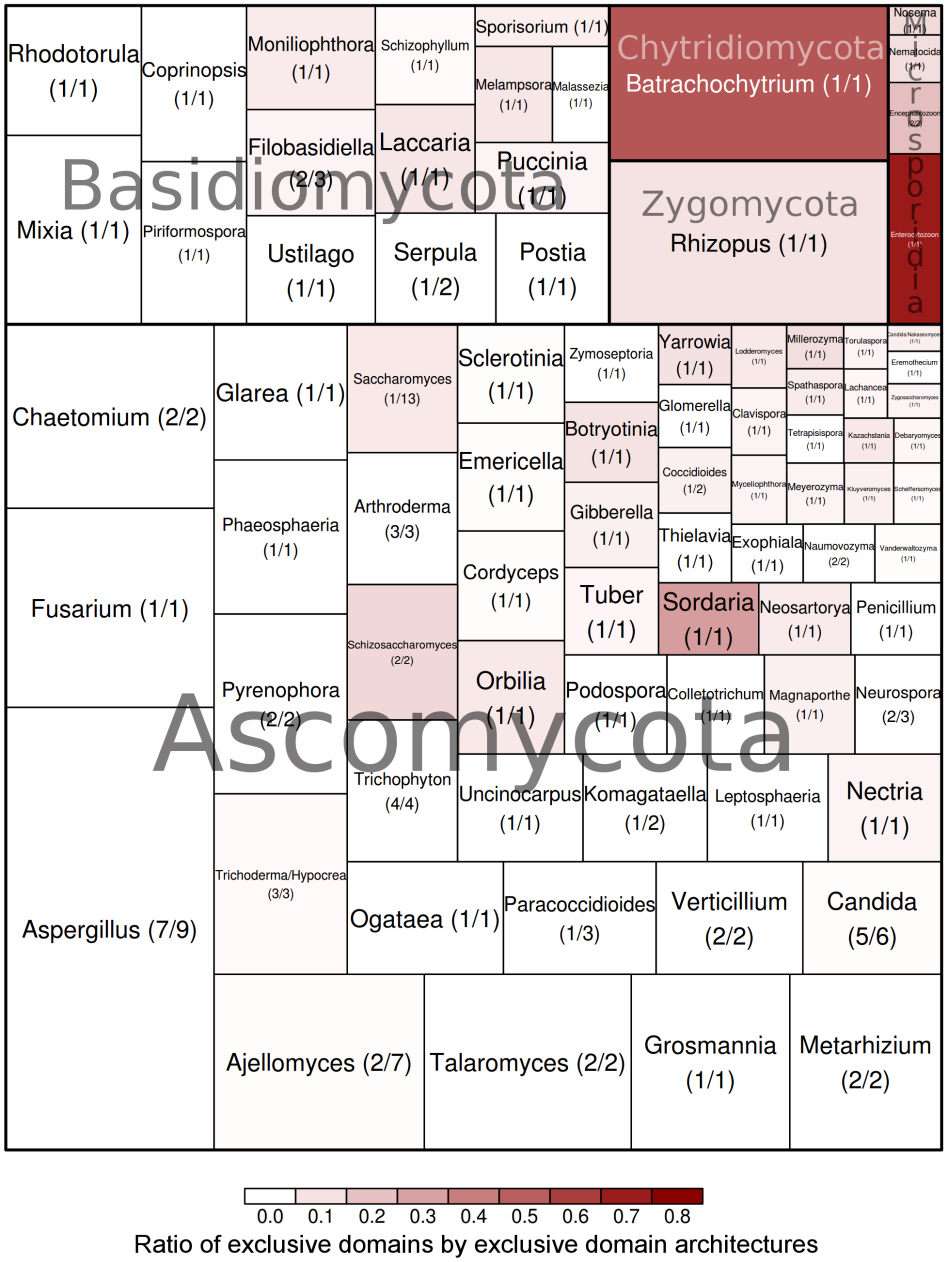
Distribution of Pfam domains and domain architectures per genus. In parenthesis, the number of species and the number of strains that belong to a given genus are indicated. The area occupied by each genus corresponds to the number of exclusive domain architectures, whereas the colour correlates with the number of exclusive domains present among those architectures (calculated as the number of domains divided by number of domain architectures).

A similar analysis of the exclusive domain architectures was done at the genus level. In this case, all the proteomes from the species belonging to the same genus were grouped (Figure 3). Domain architectures exclusive to one genus or shared by members of the same genus were considered clade-specific. Overall, 14 genera (seven of them having more than one species analysed) had more than 200 clade-specific domain architectures. *Aspergillus*, the best represented genus with 7 species and 9 strains in total, had the largest set of exclusive architectures (943), containing 104 architectures shared by at least two *Aspergillus* species (Figure 3). A list containing the results of the analysis for all genera including more than one species is included in Table 1. A comprehensive collection of the exclusive and core domains and domain architectures at different taxonomical levels (phylum, subphylum, order, genus and species) can be found in the Tables S6 and S7, respectively.

**Table 1:** List of genera including more than one species.

Genera (Nb. species/Nb. strains)	Sum of the number of strain-specific architectures	Number of genus-specific architectures	Increase (%)
*Encephalitozoon* (2/2)	20	39	95.0
*Filobasidiella* (2/3)	88	140	59.0
*Neurospora* (2/3)	59	88	49.0
*Schizosaccharomyces* (2/2)	131	152	16.8
*Trichoderma/Hypocrea* (3/3)	218	246	12.8
*Aspergillus* (7/9)	839	943	12.4
*Metarhizium* (2/2)	244	273	11.8
*Trichophyton* (4/4)	149	160	7.4
*Pyrenophora* (2/2)	230	245	6.5
*Talaromyces* (2/2)	351	372	6.0
*Candida* (5/6)	160	154	3.9
*Ajellomyces* (2/7)	367	378	3.0
*Arthroderma* (3/3)	149	145	2.6
*Chaetomium* (2/2)	387	392	1.3
*Naumovozyma* (2/2)	43	43	0

The table is sorted by the increase in the number of exclusive architectures in the genus when compared to the individual species. In parenthesis, the number of species and the total number of strains are indicated.

The information is split by taxonomical levels and allows the screening of conserved domains and domain architectures in the studied organisms. In particular, an interesting case is that of the Domains of Unknown Function (DUFs) and their related domain architectures. The specificity and exclusiveness or these domains to certain taxonomical groups could help to elucidate the underlying biological functions.

### 4. Promiscuous protein domains

Since we were interested in knowing which domains were essential for the different fungi, next we identified the list of promiscuous domains per organism, as explained in ‘Methods’. Table 2 shows a list of protein domains commonly found among the 25 top-ranked promiscuous domains in the individual organisms. Important functions for the cell survival and interaction with the environment were associated with most of the detected promiscuous domains.

**Table 2:** Protein domains most frequently found among the 25 top-ranked most promiscuous domains in all the fungal organisms.

Pfam domain name	Description	Times in top 25 ranking of promiscuous domains	Average number of bigrams	Gene ontology (GO) terms
AAA*	AAA family proteins often perform chaperone-like functions that assist in the assembly, operation, or disassembly of protein complexes	132	17	GO:0005524 ATP binding
GATase*	Glutamine amidotransferase class-I	123	7	-
SH3_1*	SH3 (Src homology 3) domains are often indicative of a protein involved in signal transduction related to cytoskeletal organization	122	11	GO:0005515 protein binding
PX	PX domains bind to phosphoinositides.	117	10	GO:0005515 protein binding; GO:0007154 cell communication; GO:0035091 phosphatidylinositol binding
PH*	PH stands for pleckstrin homology	116	9	GO:0005515 protein binding; GO:0005543 phospholipid binding
SNF2_N	SNF2 family N-terminal domain. This domain is found in proteins involved in a variety of processes including transcription regulation, DNA repair, DNA recombination and chromatin unwinding	115	12	GO:0003677 DNA binding; GO:0005524 ATP binding
Helicase_C	Helicase conserved C-terminal domain	108	20	GO:0003676 nucleic acid binding; GO:0004386 helicase activity; GO:0005524 ATP binding
MMR_HSR1	The full-length GTPase protein is required for the complete activity of the protein interacting with the 50 S ribosome and binding of both adenine and guanine nucleotides, with a preference for guanine nucleotide	98	8	GO:0005525 GTP binding
DEP*	Domain found in Dishevelled, Egl-10, and Pleckstrin (DEP). The DEP domain is responsible for mediating intracellular protein targeting and regulation of protein stability in the cell	89	5	GO:0035556 intercellular signal transduction
UBA*	UBA/TS-N domain. Found in several proteins having connections to ubiquitin and the ubiquitination pathway	88	7	GO:0005515 protein binding
TPR_1*	Tetratricopeptide repeat	86	9	GO:0005515 protein binding
zf-RING_2	Ring finger domain	80	11	GO:0005515 protein binding; GO:0008270 zinc ion binding
C1_1*	Phorbol esters/diacylglycerol binding domain (C1 domain). This domain is also known as the Protein kinase C conserved region 1 (C1) domain.	76	5	GO:0035556 intercellular signal transduction
JmjC*	The JmjC domain belongs to the Cupin superfamily. JmjC-domain proteins are hydroxylases that catalyse a novel histone modification	74	5	GO:0005515 protein binding;
UCH*	Ubiquitin carboxyl-terminal hydrolase	72	9	GO:0004221 ubiquitin thiolesterase activity; GO:0006511 ubiquitin-dependent protein catabolic process
BRCT*	BRCA1 C-terminus (BRCT) domain. Canonical BRCT phosphopeptide interaction cleft at a groove between the BRCT domains	71	7	-
PHD*	PHD folds into an interleaved type of Zn-finger chelating two Zn ions in a similar manner to that of the RING and FYVE domains	66	9	GO:0005515 protein binding
UBACT	Repeat in ubiquitin-activating (UBA) protein	65	5	GO:0005524 ATP binding; GO:0006464 cellular protein modification process; GO:0008641 small protein activating enzyme activity
TPR_2	Tetratricopeptide repeat	65	7	-
RhoGEF	RhoGEF domain. Guanine nucleotide exchange factor for Rho/Rac/Cdc42-like GTPases Also called Dbl-homologous (DH) domain. It appears that Pfam:PF00169 domains invariably occur C-terminal to RhoGEF/DH domains	57	6	GO:0005089 Rho guanyl-nucleotide exchange factor activity; GO:0035023 regulation of Rho protein signal transduction
CBM_1	Fungal cellulose binding domain	24	13	GO:0004553 hydrolase activity, hydrolyzing O-glycosyl compounds; GO:0005576 extracellular region; GO:0005975 carbohydrate metabolic process; GO:0030248 cellulose binding

Domains marked with an asterisk had been previously identified as promiscuous in animals, plants and fungi [37].

The promiscuous domain found in a highest number of organisms was the ‘ATPases Associated with cellular Activities’ (‘AAA’ or ‘AAA+’) domain, found to be promiscuous in 133 out of the 137 organisms studied. It belongs to a large and intensively studied protein superfamily. ‘AAA’ domains usually have a ring shaped oligomeric complex that conveys them several activities through the energy-dependent unfolding of macromolecules [31]. Phylogenetic analyses of ‘AAA’ proteins have suggested the existence of six main clades of ‘AAA’ domains [32], covering all kingdoms of living organisms. The second promiscuous domain most commonly found was ‘GATase’ (123 times), which is the principal component of the homonym enzyme glutamine amidotransferase, enabling the catalysis of the ammonia group from glutamine.

Interestingly, after ‘AAA’ and ‘GATase’, the most promiscuous domains identified were ‘SH3_1’ (found in 122 organisms) and ‘PX’ (117 organisms), two related domains which can interact with each other. ‘SH3 ‘domains are typically 40–60 amino acids long [33] and have a hydrophobic pocket that can bind proteins containing peptides rich in proline [34]. ‘SH3’ domains may modulate interactions with the cytoskeleton and the membrane. In contrast, the ‘PX’ domain has an average length of 130 amino acids and is characterized by: i) a PxxP motif (referenced by the ‘PX’ acronym) recognized by the ‘SH3’ domains; and ii) the basic residues that form a phospholipid binding pocket. Frequently, ‘PX’ domains co-appear with dimerization-related domains such as coiled-coil domains, increasing the affinity of the ‘PX’ domains to the cell membrane [35]. Both the ‘PX’ and ‘SH3’ domains have been identified in intracellular signalling pathways *via* protein-protein interactions [36]. Apart from the domains mentioned above, other 12 domains, including ‘PH’, ‘SNF2_N’, ‘Helicase_C’, ‘MMR_HSR1’, ‘DEP’, ‘UBA’, ‘TPR_1’ and ‘zf-RING_2’, ‘C1_1’, ‘JmjC’, ‘UCH’ and ‘BRCT’, were the ones found to be promiscuous in more than 50% of the studied fungi.

It has been previously pointed out that protein domain promiscuity is a volatile feature throughout evolution [37]. In fact, only a few domains are consistently classified as promiscuous in organisms from all major taxonomical domains. We think that this observation fits with our results since, overall, only 20 domains were found to be promiscuous in at least 25 organisms (out of the 137 studied, Table 2).

### 5. Fungal-human protein domain content comparison

So far, in this study we have performed a detailed analysis of the protein domain and architecture content of the available fungal proteomes. The resulting information can be used with different purposes in mind. In our case, we decided to further mine the data and explore approaches to detect *in silico* potential targets for antifungal agents. It is well-known that undesired side effects are one of the main issues of the currently used antifungal drugs [38], so we first aimed at finding targets that were exclusive for fungi. Since protein domains often represent different three-dimensional structures and these structures determine the binding potential of small molecules and drugs, we first tried to detect which of the domains were exclusively present in fungi and were not found in the human proteome.

**Figure 4:**
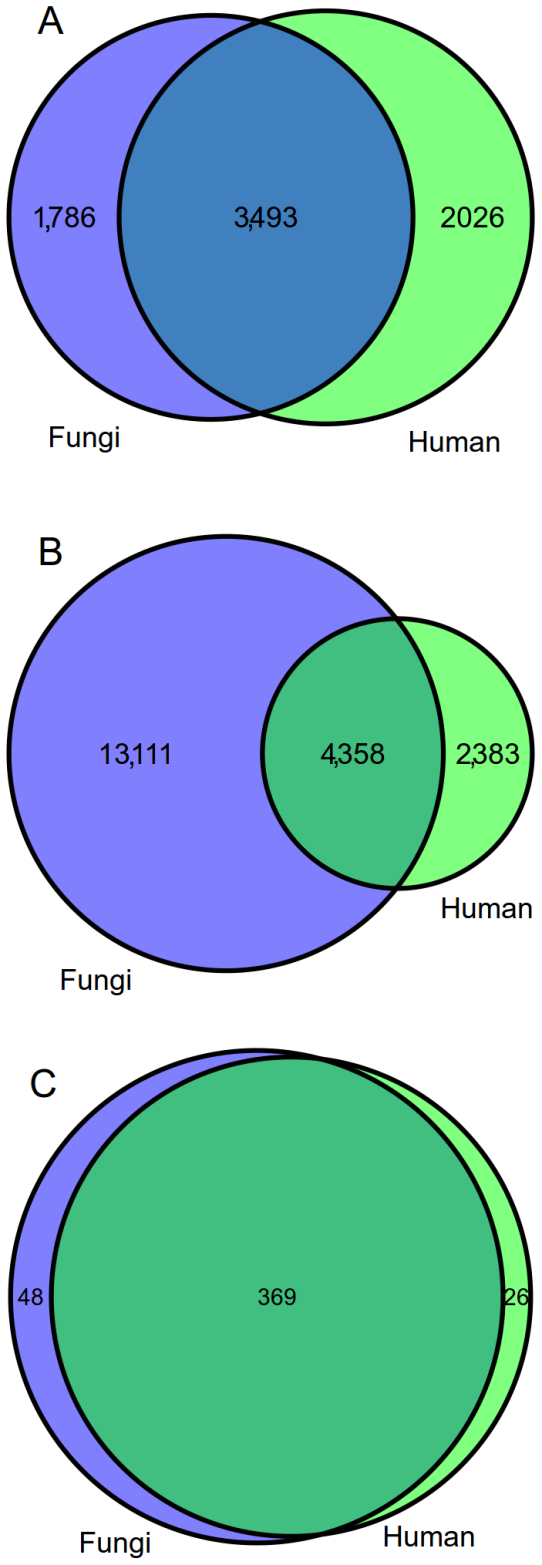
Distribution of protein domains (A), domain architectures (B) and Pfam clans (C) shared between the fungal species included in this study and *Homo sapiens*. The category “fungi” refers to the set of 137 organisms analysed.

Protein domain information of the human reference proteome (UniProtKB release 01_2013) was then analysed and compared with the information available for all the fungal organisms. At least one Pfam domain was retrieved for 72.8% of the human proteins, with an average of 2.06 domains per protein. The number of distinct Pfam domains found (5,519, 37.2% of all the Pfam domains) was slightly higher than the sum of the different domains available for all the fungal proteomes analysed (5,279). However, on the other hand, almost three times more architectures were found in the combined fungal proteomes when compared with human (17,469 vs. 6,741, respectively). The number of domains and domain architectures either exclusive or shared between human and fungi are represented in Figure 4. The number of exclusive domains and architectures for fungi were 1,786 and 13,111, respectively.

In order to have a second view on the human and fungal domain contents, we again analysed the protein domain information, but this time at the level of the Pfam domain clans. Pfam clans consist of a series of evolutionary related Pfam families which are believed to share a common ancestor [39]. Two conditions are checked to assess whether Pfam domains are grouped in the same clan: i) to have a related three-dimensional structure; and ii) to have a quality matching of the HMM (Hidden Markov Model) profiles. In total, 469 clans comprising 4,563 Pfam domains (30.7% of all the Pfam domains) were found for both human and fungi. Overall, 48 clans (10.2% of the total number of clans) were found to be composed by Pfam domains exclusive for fungi, not present in any human protein (Figure 4 and Table S8).

Based on the criterium of drug side effects, all proteins containing protein domains belonging to those 48 clans (corresponding to 77 Pfam domains) are in our opinion, potentially good initial candidates to be considered as antifungal targets. Using the already available information about the exclusive domains per taxonomic level, the list of potential targets could then be tailored to e.g. particular species, genera or phyla.

Just as one example, one of those detected 77 Pfam domains was the domain ‘DinB’ (PF05163, part of the clan CL0310), which was exclusively found in proteins from species from the genus *Aspergillus*. The ‘DinB’ domain was named after *dinB*, one member of the DNA damage-inducible *din* genes. The three *din* loci (*dinA*, *dinB* and *dinC*) were originally described in *Bacillus subtilis* as components of a global SOS-like regulatory network called SOB system [40]. It has been shown that the SOS pathway could be essential in the acquisition of bacterial mutations which lead to resistance to some antibiotic drugs [41]. This domain was only found in monodomain proteins from *Aspergillus* and might then represent a good potential target for anti-*Aspergillus* drugs.

### 6. Pathways for proteins containing fungal promiscuous domains

The second approach was to search for biological pathways that could be potential targets for antifungal compounds, focusing on proteins with promiscuous domains, since they might play a role in maintaining network stability [42]. In this case, we decided to use the identified promiscuous domains (described in section 4) that were exclusively found in fungi (described in the previous section), since promiscuous domains are believed to play a major role in cellular signalling networks. From the set of 219 fungal promiscuous domains (present in the compiled list of the 25 top-ranked for each species, Table 2), eight of them were not found in the human proteome: ‘Cas_Cas4’ (PF01930), ‘CBM_1’ (PF00734), ‘Glyco_hydro_72’ (PF03198), ‘HisKA’ (PF00512), ‘Hom_end’ (PF05204), ‘KR’ (PF08659), ‘PH_10’ (PF15411) and ‘Sirohm_synth_M’ (PF14824).

Overall, 3,675 fungal proteins in UniProtKB contained at least one of these 8 domains. We then looked for representation of these proteins in fungal pathways available in the public domain. Table 3 shows detailed information about the matched pathways in the UniPathway resource and the corresponding proteins and domain architectures found. As a result, five proteins were detected which contained one of the above mentioned eight domains, and which were represented in at least one metabolic pathway in UniPathway (Table 3). In fact, only three (out of the eight) domains were represented in those proteins: ‘CBM_1’, ‘KR’ and ‘Sirohm_synth_M’. The ‘CBM_1’ domain is a fungal cellulose-binding domain which can interact with specific structural polysaccharides [43]. The ‘Sirohm_synth_M’ domain is part of the enzyme which catalyzes the biosynthesis of siroheme, a cofactor essential for the assimilation of nitrogen and sulfur [44]. Finally, ‘KR’ is an enzymatic domain, part of the multimodular polyketide synthases, responsible of the growth of the polyketide chain.

**Table 3:** Pathways and protein domain architectures related to fungal promiscuous domains. The promiscuous domains are indicated in bold letters.

Protein(s) originally annotated	Domain architecture	Number of Proteins	Number of species	Metabolic pathway (UniPathway)	Metabolic pathway description
Q0C8M3	ketoacyl-synt∼Ketoacyl-synt_C∼Acyl_transf_1∼PS-DH∼Methyltransf_12∼KR∼PP-binding∼Condensation	4	4	UPA00875: lovastatin biosynthesis	Biosynthesis of lovastatin, an HMG-CoA reductase inhibitor produced by the fungus *Aspergillus terreus*. Lovastatin is composed of two polyketide chains
Q4WBW4; A1DBP9	Esterase_phd∼CBM_1	34	22	UPA00114: xylan degradation.	Degradation of xylan, a polymer of xylose residues
P15807; O14172	NAD_binding_7∼Sirohm_synth_M∼Sirohm_synth_C	125	96	UPA00262: siroheme biosynthesis	Biosynthesis of siroheme, the cofactor for sulfite and nitrite reductases. Siroheme is formed by methylation, oxidation and iron insertion into the tetrapyrrole uroporphyrinogen III (Uro-III)

Overall, three pathways present in UniPathway, containing these five proteins, were identified as potential targets for antifungals:

- Lovastatin biosynthesis (for the domain ‘CBM_1’). Lovastatin is commonly used as cholesterol-lowering agent. In a fungal context, it is a compound that blocks the first step of the terpene biosynthesis for the production of ergosterol (the main component of cell membranes in fungi). It was first discovered in *Aspergillus terreus* and is a precursor of other successful anti-cholesterol drugs [45]. However, the antifungal properties of some statins have been known for years and recent studies have shown that statins bring to bear relevant antifungal effects against dermatophyte fungi and, in combination with other clinically used antifungal agents, can significantly improve the treatment using combined therapy [46].
- Xylan degradation (for the domain ‘KR’). Diverse microorganisms including filamentous fungi secrete enzymes capable to digest xylan, a polysaccharide constituent of the plant cell walls. These enzymes have been used in industrial production of animal food, textiles and production of biofuels [47]. Furthermore, understanding how plants fight against fungal pathogens thanks to xylanase inhibitors could bring insights into the development of new drugs in the treatment of fungal infection in these organisms.
- Biosynthesis of siroheme (for the domain ‘Sirohm_synth_M’). Siroheme is a heme-like prosthetic group used by sulfite and nitrite reductases to convert sulfite into sulfide and nitrite into ammonia, respectively. This process is essential for the assimilation of sulfur and nitrogen by plants and consequently, for life. Sulfite reductases are found in bacteria, plants and fungi but not in animals. Assimilation of all inorganic sulfur and the majority of nitrogen in the biosphere depend on the availability of siroheme. Without it, there would be no reduced sulfur available for the synthesis of cysteine and methionine. Those amino acids are essential for animals, which are unable to reduce sulphate, and thus require to include sulfur-containing amino acids in the diet [48]. The essential role of this pathway, also described in *S. cerevisiae* [44], makes it a potential target.

Unfortunately, the amount of pathway information from fungal organisms in the public domain is quite limited at present. It was not possible to find comparable ready-to-use information in other resources apart from UniPathway (see ‘Methods’). This clearly limits the applicability of this approach at present. For instance the xylan degradation pathway may not be potentially interesting for human pathogens, but it could definitely be for plant pathogens. Therefore, independently of the three pathways detected here, we think that the described approach will be more usable as more pathway related information becomes publicly available.

Since annotation of fungal pathways in resources like UniPathway is limited to the most popular species, the protein domain architecture of the five proteins identified was searched in the rest of fungal organisms to know how common these three pathways were throughout the fungal kingdom. Assuming that proteins with the same domain architecture can have a similar function and can be involved in the same or similar pathways, the most ubiquitous pathway among the three was the “biosynthesis of siroheme”, present in 96 out of the 111 fungal species studied (Table 3). Analogous approaches can be used to determine the desired scope of a given antifungal potential.

### 7. Correlation between protein domain content and occurrence in clinical samples

The 111 fungal species used in this study were classified in five groups taking into account the frequency in which they had been found in clinical samples (see ‘Methods’ and Table 1 for the list of species and groups). Species belonging to the same group were merged and the distribution of domains and domain architectures per group was examined as before. A complete list with all exclusive domain architectures found in at least one member of each group is provided in Table S9.

Group 4 (the one including the species which are most commonly found in clinical samples) was characterized by 140 exclusive architectures. Among those, 11 architectures contained DUFs. Group 3 contained 674 unique domain architectures. The least clinically relevant groups, groups 2 and 1, had 1,801 and 3,048 architectures, respectively. They were the ones with the highest number of exclusive architectures.

When the analysis was performed at the level of the protein domains, 11 exclusive domains were found for at least one species in Group 3, whereas only two domains were found to be exclusive of Group 4. Table 4 shows the domains exclusively associated with the species of the Groups 3 and 4. Among those domains, three of them can be highlighted: ‘HI0933_like’ (PF03486), ‘TIR_2’ (PF13676), and ‘Keratin_B2_2’ (PF13885). The first one is found in redox enzymes. Redox metabolism has already been proved to be a useful target for drug development in other microorganisms [49], [50]. The ‘TIR_2’ domain belongs to a family of bacterial ‘Toll-like’ receptors. Recently, novel potential drug targets related to the ‘Toll-like’ receptor have been described in *Mycobacterium tuberculosis* [51]. Finally, the ‘Keratin_B2_2’ domain is rich in cysteine and it is present in proteins associated with keratin. These proteins belong to the same Pfam clan (CL0291) than the AMP protein, a small protein with microbial activity. Defensins from plants, insects and mammals constitute the most prominent group within the known antifungal AMPs. The number of defensin-like antifungal AMPs of fungal origin studied is increasing and they show similar structural features to the defensins. In fact, AMPs might provide to their hosts the advantage to successfully compete with organisms that possess similar nutritional and ecological requirements. It is believed that the activity of many of these fungal AMPs is based on their interaction with proteins from the cell wall or the membrane of target fungi implicated in the signalling cascade. AMP related studies constitute a promising and fast growing field, which could trigger the identification of novel related antifungal drugs in the coming years [52].

**Table 4:** Protein domains found exclusively in proteins from the Groups 3 and 4 of clinical isolates.

Pfam domain name	Domain description	Pfam clan information	Group
ATP1G1_PLM_MAT8	ATP1G1/PLM/MAT8 family	-	3
CTP_transf_3	Cytidylyltransferase. This family consists of two main Cytidylyltransferase activities: 1) 3-deoxy-manno-octulosonate cytidylyltransferase; 2) acylneuraminate cytidylyltransferase. NeuAc cytydilyltransferase of *Mannheimia haemolytica* has been characterized describing kinetics and regulation by substrate charge, energetic charge and amino-sugar demand.	CL0110: GT-A. This is the GT-A clan that contains diverse glycosyltransferases that possess a Rossmann like fold	3
FRG	FRG domain. This presumed domain contains a conserved N-terminal (F/Y)RG motif. It is functionally uncharacterised	-	3
HI0933_like	HI0933-like protein	CL0063: NADP_Rossmann. A class of redox enzymes is composed by two domain proteins. One domain, termed the catalytic domain, confers substrate specificity and the precise reaction of the enzyme. The other domain, which is common to this class of redox enzymes, is a Rossmann-fold domain	3
PTS-HPr	PTS HPr component phosphorylation site	-	3
SdiA-regulated	SdiA-regulated. This family represents a conserved region approximately within a number of hypothetical bacterial proteins that may be regulated by SdiA, a member of the LuxR family of transcriptional regulators. Some family members contain the Pfam:PF01436 repeat	CL0186: Beta_propeller. This large clan contains proteins that contain beta propellers. These are composed of between 6 and 8 repeats. The individual repeats are composed of a four stranded sheet	3
Sugarporin_N	Maltoporin periplasmic N-terminal extension. This domain would appear to be the periplasmic, N-terminal extension of the outer membrane maltoporins	-	3
TIR_2	TIR domain. This is a family of bacterial Toll-like receptors	CL0173: STIR. Both members of this clan are thought to be involved in TOLL/IL1R-like pathways, by mediating protein-protein interactions between pathway components. The N-termini of SEFIR and TIR domains are similar, but the domains are more divergent towards the C-terminus	3
Uma2	Putative restriction endonuclease. This family consists of hypothetical proteins that are greatly expanded in cyanobacteria. The proteins are found sporadically in other bacteria. A small number of member proteins also contain Pfam:PF02861 domains that are involved in protein interactions. Solutions of several structures for members of this family show that it is likely to be acting as an endonuclease	CL0236: PDDEXK. This clan includes a large number of nuclease families related to holliday junction resolvases	3
FixP_N	N-terminal domain of cytochrome oxidase-cbb3, FixP. This is the N-terminal domain of FixP, the cytochrome oxidase type-cbb3. The exact function is not known	-	3
MFMR	G-box binding protein MFMR. It is between 150 and 200 amino acids in length. The N-terminal half is rather rich in proline residues and has been termed the PRD (proline rich domain), whereas the C-terminal half is more polar and has been called the MFMR (multifunctional mosaic region). It has been suggested that this family is composed of three sub-families called A, B and C, classified according to motif composition	-	3
HEPN	HEPN domain	CL0291: KNTase_C. This alpha helical domain is found associated with a variety of nucleotidyltransferase domains	4
Keratin_B2_2	Keratin, high sulfur B2 protein	CL0520: Keratin_assoc. Families in this clan are cysteine-rich and are from proteins associated with Keratin	4

Extended information about all domains exclusively found in different at least one species from the described groups according to their occurrence in clinical samples can be found in Table S10. With this approach we aimed at looking for drug targets in some of the most common human pathogens. However, the classification used here has clearly some limitations. The main drawback is that it is based on two epidemiological studies performed only in Spain. Although some worldwide information was also taken into account to perform the classification, the scenario can be slightly different in other parts of the world. However, the spectrum of species isolated in the Spanish studies is comparable with other studies performed in other countries (such as the TransNet study in the US). It is also important to highlight that both studies are focused mainly in deep infections and therefore the highly prevalent subcutaneous infections could not be classified according to these criteria. However, this last issue can be assumed since the focus in the development of new antifungal drugs is usually put in the type of diseases where the mortality rate is particularly high and where multi-resistant fungi are mainly present.

### 8. Domain ligand binding

As a third approach to identify potential targets for drugs, the list of protein domains produced by Kruger and colleagues in a previous study [21] was used to assess the ligand-binding capability for each of the fungal domains. They generated a list of 215 Pfam domains where some evidence of ligand-binding capabilities to small molecules was found.

When we compared directly this list with the generated list in the previous section (according to the occurrence of the fungi in clinical samples), we found that none of the exclusive domains from proteins of the Groups 3 or 4 were identified to have small-molecule binding potential. Only seven domains exclusive for organisms belonging to the less relevant group 2 appeared to have small-molecule binding potential: ‘BH3’ (PF15285), ‘Cons_hypoth698’ (PF03601), DUF2146 (PF10220), ‘Gb3_synth’ (PF04572), ‘NCD2’ (PF04905), ‘bact-PGI_C’ (PF10432) and ‘TTKRSYEDQ’ (PF10212). Interestingly, these exclusive domains were present mainly in proteins of the multi-resistant species *F. oxysporum* and *R. delemar* (closely related to *Rhizopus oryzae*).

Based on the structural similarities, we decided to include in the comparison all the Pfam domains belonging to the same clans than the list of 215 domains initially studied (extending the initial number to 1,193).

As a result of this second analysis, three domains were exclusively found in proteins exclusive of species of the Group 3: ‘CTP_transf_3’ (PF02348), ‘HI0933_like’ (PF03486, also highlighted in the previous section) and ‘Uma2’ (PF05685). ‘CTP_transf_3’ is a domain found in cytidylyl-transferase membrane proteins, which are important regulatory enzymes in the synthesis of phospholipids in eukaryotic cell membranes [53]. As mentioned before, the ‘HI0933_like’ domain can be found in redox enzymes. It belongs to the ‘NADP_Rossmann’ clan whose domains typically bind nicotinamide adenine dinucleotide (NAD+) [54]. Finally the ‘Uma2’ domain family, frequently found in bacteria and archaea, is believed to be part of restriction endonucleases, which are enzymes which cleave DNA and allow DNA recombination [55]. ‘CTP_transf_3’ (unlike ‘HI0933_like’ and ‘Uma2’) was found in human proteins. The ‘HI0933_like domain’ was found only in one protein (Q0CDT4) of *A. terreus* whereas ‘Uma2’ was exclusively identified in *Trichophyton rubrum* (protein F2SXP0). Both are putative uncharacterized poorly annotated proteins. Q0CDT4 was predicted to have a signal peptide using SignalP [56], indicating that it could be secreted. To summarize, these three domains have potential ability to bind small molecules and domains from the same clan have been found exclusively in fungal organisms with clinical interest, so they could be further studied as potential antifungal targets.

### 9. Overall discussion

In this study, we have characterized the protein domain and domain architecture content of the available fungal proteomes (including the phylum Microsporidia) and we have shown how that information can be used *in silico* to detect potential candidate targets for antifungal drugs. Throughout the study, we have considered and taken into account information coming from both individual domains and complete protein domain architectures. In the case of the latter, independent architectures were defined as those ones in which the order or number of domains were different (taking into account domain duplications).

In analogous previous studies, protein domain order and domain repetitions were considered in a different way, assigning the same domain arrangement regardless of the number of consecutive copies of a single given domain [10], [13], [37], [57]. That means that some of the results reported here cannot be directly compared with the ones reported previously. We decided to follow this approach since recent studies had noted the importance of domain recurrence (repetitions of the same domain within a protein) in the overall domain function [58]. Furthermore, domain repetition is a predominant mechanism for protein diversity and evolution [59]. For instance, in some cases it has been shown that the repetition of a domain in a multidomain protein is essential for the protein function. One good example is the glutamate receptor interacting protein (GRIP) consisting in 7 ‘PDZ’ domains, two of them interacting with an α-amino-3-hydroxy-5-methyl-4-isoxazolepropionic acid (AMPA) receptor, but only in the presence of the adjacent copies [58]. Another example is the existence of the multi-modular enzymes non-ribosomal peptide synthetases (NRPS) and polyketide synthases (PKS) [60], [61], which are responsible of the synthesis of multiple biologically active products produced by bacteria and fungi, among other organisms. The number and exact order of these domains will determine the resulting synthesized compound/s.

Another reason for the consideration of the architectures as they were, is that part of the approach developed in this project is planned to be used to improve automatic protein sequence annotation in UniProtKB [62]. UniProtKB has two sections: UniProtKB/Swiss-Prot, which is manually annotated and reviewed, and UniProtKB/TrEMBL, which is automatically annotated using different bioinformatics approaches. The general method used to annotate proteins coming from new genomes is to transfer the annotation from homologous proteins identified by sequence-based or protein domain-based approaches. It is also possible to connect the information of the domain architectures specific to a given taxonomical level with the detected promiscuous domains. This information could be used to retrieve more information on the unknown functions of certain domains.

For example, knowing that the fungal promiscuous domain ‘PX’ is usually involved in targeting proteins to cell membranes, domain architectures including ‘PX’ domains might be of interest. Looking into this particular case, we identified the domain architecture ‘PXB∼PX∼DUF3818’, present in 106 proteins of all fungal phyla. The only protein manually annotated in UniProtKB with this domain architecture is Q06839, a peripheral membrane protein believed to be involved with cell communication process (GO:0007154) in *S. cerevisiae* (strain ATCC 204508/S288c). From this observation two applications of the work presented here could take place: on one hand, proteins containing the same domain composition could be similarly annotated. On the other hand, the importance of these particular proteins and the corresponding domains (like DUF3818) could be further investigated given their membrane localization and the important cell communication process in which these proteins are believed to play a role.

As a result of this comprehensive study, we provide access to the full list of ‘core’ and exclusive domains and domain architectures at different levels of the taxonomic classification and also identify the promiscuous domains. This information can be a very valuable resource for researchers interested in comparative studies between different fungal organisms. Here, we have only highlighted some examples of how this information could be used, but it is clear for us that more focused studies could be performed on particular groups of organisms, using all the generated information here. This information could also be combined with genome features such as gene clusters (very frequently found in fungi, e.g. for genes involved in secondary metabolism) or synteny.

However, in this study we decided to focus on the possible application of this information in the detection of antifungal targets. We then followed three different approaches. First of all, we identified those protein domains and domain architectures that were present in fungi but not found in the human proteome. Secondly, using the promiscuous domains, we identified three pathways whose components could be targeted. Last, we created five groups of organisms depending on their occurrence in clinical samples and then inferred small-molecule protein domain binding information obtained in a recent study involving small molecules stored in the ChEMBL database. The results coming from these three approaches constitute just a first step and should be taken with caution, since they have different inherent limitations. It is also expected that these approaches will provide new information when new data (e.g. pathway related information) is made available in the public domain.

Analogous studies where the interaction between protein domains and small molecules is assessed, are becoming more popular in the last few years. For instance, recently, using data from Protein Data Bank (PDB) structures, more than thirteen thousand physical interactions between small molecules and protein domains were identified [63]. The authors found that the capability of the protein domains to bind particular small molecules did not depend only on the protein sequence and protein structure. For example, protein domains distributed in different domain families and biological pathways were able to bind the same or similar small molecules. Some very heterogeneous domains were able to bind hundreds of small molecules. In addition, they found that 12% of the small molecules were able to bind multiple domains. It is also important to highlight that it is envisioned that small-molecule and protein domain binding information could be used in the future for finding the optimal combination of drugs in treatments.

Throughout this study we have used domain information coming from Pfam-A domains, so all the conclusions are limited to that context. One fact to consider is that protein sequence coverage in Pfam for fungi is lower than for other organisms, especially for species like *M. laricis-populina* (coverage of 36.5%) and *P. graminis sp. tritici* (coverage of 39.5%). In the future, this study could be extended by using Pfam-B domains and protein domain information coming from other databases, for instance from other protein domain/family resources that are also part of the InterPro consortium [64]. However, integrating protein domain information obtained using different computational methods or coming from different resources makes direct comparisons troublesome. In addition, another way to continue this study would be to use the sequences that currently lack protein domain annotation as an input to perform local protein sequence alignments with different methods and thresholds, and then generate the corresponding HMM-based profiles, in order to increase the detectability of homologous proteins.

Overall, this manuscript provides a comprehensive analysis of protein domain and domain architectures in the available fungal proteomes and shows three approaches that can be used as a first step in the detection of new antifungal targets. These approaches could also be used for organisms with clinical interest other than fungi e.g. bacteria. Therefore, analogous analyses could be performed for different groups of pathogenic bacteria using as a starting point the scripts provided (available at https://github.com/alexbarrera/fungidomDB). We hope that some of these strategies can be refined and applied in the practice, followed by *in vivo* validation.

## Methods

### 1. Proteome sets and domain information

The proteomes used in this study were obtained from UniProtKB (http://www.uniprot.org/) [62], release 2013_01. Only fungal complete and reference proteomes were included. In total, 137 fungal proteomes belonging to 111 species were analysed. All complete proteomes available for species with more than one strain (12 species) were also added to the collection (Table S1). The human “reference proteome” (including protein isoforms) was also retrieved for comparison purposes (64,677 proteins). “Reference proteomes” in UniProtKB are defined as complete proteomes targeted for manual annotation by curators, to emphasize the reliability of their annotations.

Domain information was obtained from Pfam [7] release 27.0 (June 2012). The Pfam database is divided in a collection of manually curated families known as Pfam-A and a set of automatically generated families named Pfam-B. Pfam-A domains and Pfam clans were considered. The mapping of Pfam domains to proteins was obtained *via* FTP (ftp://ftp.sanger.ac.uk/pub/databases/Pfam/releases/Pfam27.0/). Only 425 protein sequences (0.035%) from UniProtKB release 2013_01 were not present in Pfam 27.0. For those proteins, Pfam domain information was obtained directly from UniProtKB using a tailored extended version of UniProtJAPI, the Java Application Programming Interface (API) to access UniProtKB [65].

Additional functional annotation for Pfam domains was retrieved using Gene Ontology (GO) terms (http://www.geneontology.org) [66]. Information available in the file ‘*Pfam2GO.txt*’ (version 2013/07/24) was used to map GO terms to the Pfam domains. These expert manual annotations are originally assigned by curators in the InterPro team based on the function of particular domains rather than the function of domain families [67].

A local MySQL database was developed to store the protein sequence and domain information. The analysis of domain and architectures was performed using R (http://www.r-project.org) version 2.15.3 and scripts developed in Python. The charts and diagrams were produced using the following R packages: ‘ggplot2’ [68] for the histograms and ‘treemap’ [69] to generate the distribution of domains and architectures per genus. Finally, standard functions were used to plot the pie charts and Venn diagrams. All the scripts used in this study and related documentation are available at https://github.com/alexbarrera/fungidomDB.

### 2. Fungal taxonomy

The nomenclature of the fungal organisms followed in this study was the same one used in UniProtKB (release 01_2013). The taxonomical classification used was the one provided by the National Centre for Biotechnology Information (NCBI) [70]. One limitation of the NCBI taxonomy is that not every taxonomical category (e.g. phylum, subphylum, genus, etc) is defined for every species. In that case, the strategy followed was to propagate the parent taxonomical level in those taxonomical categories that were lacking a specific one. Of note, *P. brasiliensis* Pb01 has been recently reclassified as *P. lutzii* [27], [28]. However, we kept the original nomenclature used in UniProtKB to facilitate the traceability and reproducibility of the results.

### 3. Analysis of the promiscuity of protein domains

A method to measure the weighted bigram frequency (WBF), introduced by Basu and colleagues [37], was used to perform the analysis. This method is an adaptation of the Kullback-Leibler information gain formula. To reduce the impact of the over-abundance of domains, this algorithm divides the number of distinct domain combinations by the frequency in which the domain is found in the proteome. The rationale behind is to minimize the impact of highly abundant domains that would otherwise account for the majority of the distinct domain combinations.

Information in Table 2 was computed applying this metric for each domain in each organism. To consider one domain as promiscuous, its WBF value had to be greater than the WBF value of a domain appearing only one time in the given proteome in combination with another domain. Promiscuous domains were ranked according to their ‘promiscuity’.

### 4. Pathway related information

Metabolic pathway information was retrieved from the public resource UniPathway (http://www.unipathway.org/) [71]. The mapping between the UniProtKB entries and the UniPathway pathways was obtained from UniProtKB (release 07_2013). UniPathway was chosen over other pathway resources as KEGG (http://www.genome.jp/kegg) or MetaCyc (http://metacyc.org) due to the comprehensive mapping to UniProtKB entries and the hierarchical representation of this information. In addition, entities from UniPathway are cross-linked to other major pathway resources such as KEGG, MetaCyc and ‘The SEED’ (http://www.theseed.org).

### 5. Classification of the fungal organisms according to their occurrence in clinical samples

Based on the frequency of appearance in clinical samples (according to two recent epidemiological prospective studies carried out in Spain [72], [73]), the fungi were classified in five groups using the following criteria (Table S1):

- Group 4: Species with more than 100 isolates in any of the previously cited epidemiological studies.
- Group 3: Species with more than 50 isolates in the study carried out by Puig-Asensio *et al.* [72], or more than 20 isolates in the work published by Alastruey-Izquierdo and colleagues [73], or frequently isolated in other regions (according to the data described in http://www.life-worldwide.org/).
- Group 2: Species isolated at least once in any of the previously cited epidemiological studies.
- Group 1: Species not isolated in the cited epidemiological studies, but present in clinical samples of the collection of fungal strains from the National Centre for Microbiology (Spain).
- Group 0: Neither isolated in the two epidemiological studies cited nor present in the Spanish collection of fungal strains from the National Centre for Microbiology.

### 6. Analysis of small molecule potential binding to protein domains

A list of *in silico* predicted protein domains that can bind small molecules was collected from a recent study [21]. The authors explored the binding capability of ligands and small molecules included in the ChEMBL database [22] to Pfam domains. The resulting list of domains (available in http://www.ebi.ac.uk/~fkrueger/mapChEMBLPfam/) was used to cross-check this capability for the fungal domains included in this study.

## Supporting Information

Figure S1 **Number of domains and domain architectures for each species, grouped by subphylum.** From top to bottom, species and strains are sorted in descending order according to the proportion of domain architectures per domain. Endemic dimorphic fungi are marked with an asterisk.
(PNG)

Table S1 **Fungal species overview.** Excel file containing a detailed list of the species and strains used in this study, along with their basic proteome information.
(XLS)

Table S2 **Core domains and domains found in single and multidomain proteins.** Excel file containing the exclusive domain lists for single and multidomain proteins. The information is split in five tabs: (i) core domains found in all species; (ii) exclusive domains in single domain proteins; (iii) exclusive domains in multidomain proteins; (iv) domains found in both single and multidomain proteins; and (v) core domains found in all species without including Microsporidia species in the analysis.
(XLS)

Table S3 **Core domain architectures.** Excel file containing the core domain architectures represented in every organism analysed. The first tab contains the core domain architectures found in all species whereas the second one shows the core domain architectures in all fungal species without including Microsporidia species in the analysis. Notice that there is an entry for each distinct domain found in a multidomain domain architecture.
(XLS)

Table S4 **Exclusive domain architectures per species.** Tab-delimited file listing the Pfam domains architectures and fungal species in which those domain architectures were found exclusively.
(CSV)

Table S5 **Exclusive domains per species.** Tab-delimited file listing the Pfam domains and fungal species in which those domains were found exclusively.
(CSV)

Table S6 **Exclusive and core domains by taxonomical categories.** Tab-delimited file listing the Pfam domains that are found to be exclusive at least in one fungal phylum. Columns named after a taxonomical level (phylum, subphylum, order, genus and species) contain a value different than 0 if the corresponding domain was only found in proteins from such taxonomic level. Additionally, for each category, a column named *core* with a value of 1 indicates the presence of that domain in proteins of all species under that given category.
(CSV)

Table S7 **Exclusive and core domain architectures by taxonomical categories.** Tab-delimited file listing the Pfam domain architectures that are found to be exclusive at least in one fungal phylum. Columns named after a taxonomical level (phylum, subphylum, order, genus and species) contain a value different than 0 if the corresponding domain architecture was only found in proteins from such taxonomic level. Additionally, for each category, a column named *core* with a value of 1 indicates the presence of that domain architecture in proteins of all species under that given category.
(CSV)

Table S8 **Clans containing fungal domains but no human domains.** Excel file listing the 48 clans for which no Pfam domain identified in a human protein was associated.
(XLS)

Table S9 **Domain architectures exclusive by clinical groups.** Tab-delimited file listing the exclusive domain architectures per clinical isolate.
(CSV)

Table S10 **Domains exclusive by clinical groups.** Tab-delimited file listing the exclusive domains per clinical isolate.
(CSV)
