# Supplementary figures and images for: Analysis of the Protein Domain and Domain Architecture Content in Fungi and Its Application in the Search of New Antifungal Targets
Source: PLoS Comput Biol. 2014 Jul 17;10(7):e1003733. doi: 10.1371/journal.pcbi.1003733 (PMC4102429; doi:10.1371/journal.pcbi.1003733)

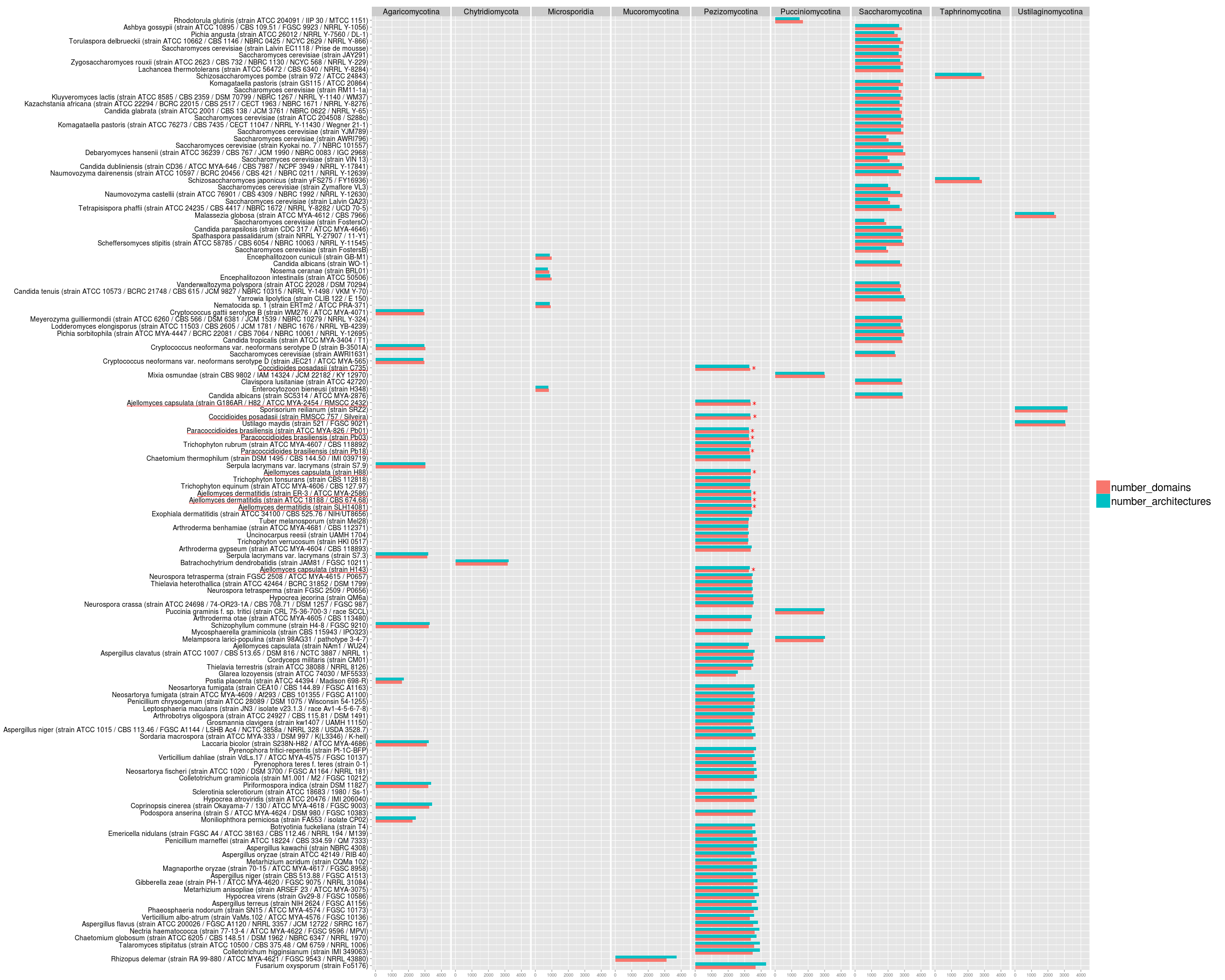

Supplement: Figure S1 — Number of domains and domain architectures for each species, grouped by subphylum. From top to bottom, species and strains are sorted in descending order according to the proportion of domain architectures per domain. Endemic dimorphic fungi are marked with an asterisk. (PNG) [file pcbi.1003733.s001.png]
